# Recognition: key to the entrepreneurial strategies of rural coalitions in advancing access to health care

**DOI:** 10.1186/s12939-019-1021-3

**Published:** 2019-07-30

**Authors:** Kathy L. Rush, Mike Chiasson, Mary Butterfield, Silvia Straka, Barbara Jean Buckley

**Affiliations:** 1 School of Nursing, University of British Columbia Okanagan, ART 150 - 1147 Research Road, Kelowna, British Columbia V1V 1V7 Canada; 2 Faculty of Management, UBC-Okanagan, EME 4159-1137 Alumni Avenue, Kelowna, B.C. V1V 1V7 Canada; 3 Faculty of Management, UBC-Okanagan, EME 4103-1137 Alumni Avenue, Kelowna, B.C V1V 1V7 Canada; 4 School of Social Work and Human Service, Thompson Rivers University, 805 TRU Way, Kamloops, BC V2C 0C8 Canada; 5 School of Nursing, Thompson Rivers University, 805 TRU Way, Kamloops, BC V2C 0C8 Canada

## Abstract

**Objectives:**

Considerable evidence has advanced the role of citizen-led coalitions (CLC) in supporting the health and social needs of rural citizens. There has been little research focusing on the experiences and strategies of coalitions, with their limited resources and status, in targeting health inequities in their rural communities. The aim of this study was to understand the entrepreneurial strategies and experiences of rural coalitions to effect change in the delivery of health services for their older adult populations.

**Method:**

A qualitative descriptive study method was used to generate understanding of the entrepreneurial experiences and strategies of CLCs in advancing health services to meet the health and social needs of their citizens. Seven diverse CLCs (*n* = 40) from different rural communities participated in focus groups and in individual and coalition-level surveys. Thematic analysis was used to construct themes from the data.

**Results:**

Two over-riding themes emerged: entrepreneurial strategies and societal recognition. CLCs engaged in numerous entrepreneurial strategies that enabled actions and outcomes in meeting their health care needs. These strategies included: securing quick wins, leveraging existing resources, and joining forces with stakeholder groups/individuals. However, despite these strategies and successes, coalitions expressed frustration with not being seen and not being heard by decision-makers. This pointed to a key structural barrier to coalition successes -- a broader societal and institutional problem of failing to recognize not only the health needs of rural citizens, but also the legitimacy of the community coalitions to represent and act on those needs.

**Conclusions:**

Despite the potential for coalitions to mobilize and effect change in addressing the inequities of rural health service access for older adults, broader barriers to their recognition, may undermine their entrepreneurial strategies and success.

## Background

### Equitable access to care

Well-known health and health care disparities exist in rural communities in Canada [1, 2]. Obesity, cardiovascular disease, diabetes, and hypertension along with lower life expectancy are known to be higher in rural than urban areas [3]. Despite their greater health care needs, rural residents often have less health care access [2]. Rural health service delivery is challenging due to diseconomies of scale, the small client base, and the remoteness from specialist services in smaller and less densely populated areas [4].

Access to quality care “without financial or other barriers” has long been a central goal of Canadian health care [5]. However, access to health care is not necessarily satisfied by access to basic services or by the provision of government-subsidized universal health care coverage, and understanding it in these simplistic terms may obscure larger issues of equity and social justice. Access and its provision is not a singular outcome, and health care access may be better understood in terms of responsiveness to *need*, with needs spanning differences across individuals and communities, across regions and geography, across class, race and gender. In a survey [6] of the Canadian healthcare system, 38% of Canadians viewed equal access to health care services as the most important aspect of health care but 14% responded they would be unable to access the appropriate services.

Challenges related to rural health services access have been well documented [7]. Despite contradictory and inconclusive comparative evidence of health care access between rural and urban populations [8], health service inequities across rural-urban regions have been reported. A Canadian survey of health care utilization patterns revealed significantly greater proportions of rural residents reporting receipt of hospital care in the previous 12 months and with a significantly higher risk of hospitalization (10 to 27%) compared to their urban counterparts [9]. In their study assessing the equity of health care services, Loftus et al. [10] found that 24% of the rural population reported no use of preventive care in the past year, compared to just under 19% of the urban population. Further, rural communities have reported a shortage of primary care physicians for many years and have felt this chronic shortage longer and more severely than have urban areas [11].

### Strategies for improving access to care: community action and health coalitions

A literature review found many innovative and evidence-based initiatives for delivering services to rural and remote communities [12]. Common among the studies was a strong need for local capacity development and community-led programming. One such capacity development comes from citizen-led coalitions (CLCs) which have increasingly formed to address common crises in many rural communities, such as hospital closures or provider shortages.

Citizen-led coalitions can be viewed as democratic publics or voluntary groups that form as a result of citizens sharing the consequences of an identified social problem [13]. One of the key roles of community coalitions is representing service users who are often marginalized, hidden or, ignored [14–16] and ultimately lack recognition. The role of coalitions in bringing recognition to marginalized groups has been implicit and tangential in the literature. In a case study of the strategic role of third-sector agencies, “being at the top table” (pg. 227) was critical in developing a strong third sector and user presence [16]. Third sectors were viewed as bringing something extra, such as volunteers and access to funding.

### Coalition success and functioning

Emphasis in the coalition literature has been on coalitions and their effects, or lack thereof, at producing tangible results, and less on their conditions and strategies [15, 17]. Despite a plethora of studies related to the use of coalitions in addressing health promotion and disease prevention, little is known about how community coalition strategies in rural communities help to meet essential health services for their citizens [18]. Key to citizen-led coalitions, a form of social enterprise and community co-production in rural settings [19], are the so-called social entrepreneurial strategies of the individuals and groups in the coalition. Broadly defined, these social entrepreneurial strategies foster, directly or indirectly human, material and cultural resources necessary for institutional and industrial change. More specifically, to bring about such change, social entrepreneurial strategies involve the identification and combination of resources to produce novel outcomes [19].

Within this entrepreneurial view, coalitions face a number of challenges in advocating for their communities, among them a lack of access to key resources, such as rural-based data to present facts, provide statistics, and identify gaps. In addressing these challenges, much of the existing health coalition literature either focuses on coalition capacities for effectively making change within communities or specific spheres, or the capacities that make coalitions themselves effective [18]. However, little research explicitly examines the experiences and strategies of coalitions, with their limited resources and status, in targeting health inequities. The emergence and existence of health coalitions have been implicitly linked to inequitable distribution of health services and resources [20]. The purpose of this study was to understand the entrepreneurial experiences and strategies of rural coalitions to effect change in the delivery of health services for older adult populations within their communities.

## Methods

### Design

The study used a qualitative descriptive approach [21] to understand the entrepreneurial strategies of community-lead coalitions in their attempts to increase rural health service delivery. Qualitative description was used to produce a detailed, and nuanced interpretation that stayed close to the data and the participants’ everyday language [21]. Consistent with the constructivist paradigm, which views reality as socially constructed, it emphasized a bottom-up approach engaging coalitions in describing their experiences and strategies in meeting local health and social needs.

### Sample

Recruitment commenced following joint ethics approval from two western Canadian universities and a large regional health authority (H16–01398;101263). Recruitment occurred through a two-step process, initially at the rural coalition level, and then at the individual coalition member level. Coalition level recruitment occurred through an invitation from the research lead of a regional health authority who was offering research support for rural community initiatives. All coalitions that responded to this initial invitation were subsequently invited to participate as community partners in the research study. Coalition group leads circulated a letter of invitation to their members to participate in a focus group as part of the research study. In addition to the letter of invitation, coalition leads also circulated to their members a consent form, a demographic form, a focus group discussion guide, and focus group details (date, time, location).

### Data collection

Multiple sources of data were collected – focus group interviews, individual demographic information, coalition surveys, and field notes. Focus groups were conducted with seven of eight coalitions initially invited to participate; one coalition that initially responded was unable to participate due to scheduling challenges. Following consent, focus group participants completed demographic forms that asked questions about age, sex, occupation, education, marital status, length of time in community and in the group. Additionally, each coalition lead was asked to complete a survey that asked factual questions about the coalition’s location, history, structure, goals, and funding. Recorded focus groups, which were approximately 1 ½ hours in duration, built on the survey using a semi-structured interview guide to elicit the coalitions’ experiences and narrative accounts of their history, evolution, processes used for decision making and priority setting, enablers, barriers, health service partnerships, and accomplishments. One research team member facilitated the focus groups while other team members (*n* = 1–5) asked follow-up questions, kept notes of the sessions, and ensured contextual data were being obtained. Having at least two research team members made data collection more manageable, especially with larger groups, and allowed for the cross-checking of data and findings.

### Data analysis

Data were transcribed verbatim and analyzed using Morse and Richards’ [22] thematic analysis framework consisting of three processes: (i) topic coding, (ii) creating categories and (iii) abstracting or conceptualizing. Initially, data were open coded, involving careful line-by-line reading of each transcript for possible meaning. Units of meaning, consisting of words, phrases, sentences or paragraphs were used to describe coalitions’ strategies and perceived effects of those strategies. An important part of coding the data was the identification of strategies that attracted and influenced other stakeholders, such as: citizens, health care workers, policy makers, health authorities, and government. Similar codes were clustered to generate themes, which became the initial coding schema used in NVivo 9™ to code within and across transcripts. Continual refinement of the coding schema occurred until all units of meaning had been categorized. Recurring themes emerged suggesting that data saturation had been achieved. To ensure that the themes arising from the analysis reflected the coalitions experiences, strategies and current realities, findings were shared with participants in a planning meeting that brought coalition representatives together. Coalitions consented to written recording of the planning meeting discussions. These data were added to NVivo and became part of the iterative analysis.

### Findings

The sample consisted of seven rural CLCs (*n* = 40) formed to advance rural health services for older adults in their respective communities. A description of the sample appears in Table 1. Coalitions varied from a loose, informal structure to a formal governance structure. Most coalitions had been formally founded from 2 to 6 years ago but they all described “searching for health care for a very long time.” Memberships ranged from 3 to 300 members, often a combination of active and inactive members, and with variable membership arrangements. Participants had a mean age of 64 years (range: 28–85 years), generally representing the community coalition’s membership, and included a predominance of older adults (62%).

**Table 1:** Older Adult Participants’ Demographics and Information – Collective Group (*N* = 40)

Variable	N	Mean	SD	Range
Age (years)	38	64.63	13.67	28–85
Years lived in community	40	18.26	11.16	2.5–49
Years as member of group	31	3.40	2.72	1–14
Variable	Category	Participant Number	% of Focus Groups	
Community group	Coalition A	4	10.0	
	Coalition B	6	15.0	
	Coalition C	11	27.5	
	Coalition D	3	7.5	
	Coalition E	5	12.5	
	Coalition F	8	20.0	
	Coalition G	3	7.5	
Identified gender	Male	9	22.5	
	Female	31	77.5	
Marital status	Married/common law	29	72.5	
	Widowed	3	7.5	
	Divorced	7	17.5	
	Other	1	2.5	
Occupation (multiple answers allowed)	Retired	22	55.0	
	Semi-retired	5	12.5	
	Self-employed	7	17.5	
	Part-time work	5	12.5	
	Full-time work	5	12.5	
	Farmer	1	2.5	
	Other	2	5.0	
Highest educational attainment	Partial high school	1	2.5	
	High school graduate	2	5.0	
	Vocational training	3	7.5	
	Partial college/university	8	20.0	
	College graduate	6	15.0	
	University graduate	12	30.0	
	Post graduate degree	7	17.5	
	Other	1	2.5	
Living situation	Live alone	7	17.5	
	Live with partner/spouse	31	77.5	
	Live with a family member(s)	1	2.5	
	Live in a senior’s residence	1	2.5	
Time to drive from home to a community with a hospital	1 h or less	34	85.0	
	3 h or more	1	2.5	
	Other	3	7.5	
	Missing	2	5.0	
Time to drive from home to emergency/acute services	Less than 30 min	14	35.0	
	30 min to an hour	12	30.0	
	1 to 2 h	11	27.5	
	Missing	3	7.5	
Time to drive from home to health management/maintenance services	Less than 30 min	18	45.0	
	30 min to an hour	10	25.0	
	1 to 2 h	6	15.0	
	2 to 3 h	1	2.5	
	Missing	5	12.5	
Ongoing medical support and services of a primary care physician	Yes	18	45.0	
	No	10	25.0	
	Missing	6	15.0	
Ongoing medical support and services of a medical specialist	Yes	17	42.5	
	No	18	45.0	
	Missing	5	12.5	
Became involved with group to improve accessibility and availability of local health care	Yes	34	85.0	
	No	3	7.5	
	Missing	3	7.5	
Plan to actively participate in group in future	Yes	32	80.0	
	No	2	5.0	
	Missing	6	15.0	
Involved in other volunteer strategies	Yes	29	72.5	
	No	6	15.0	
	Missing	5	12.5	

### Themes

The coalitions profiled the strengths of their local health and social services, such as lab and transportation services and age-friendly initiatives. Yet aging rural populations and ongoing physician recruitment and retention issues continually challenged them to address perceived inadequacies in services. Such inadequacies compelled coalitions to actively work toward ensuring the availability of close-to-home health care services (e.g., primary care) in their communities. It is within this active work of the coalitions that two over-riding themes emerged: entrepreneurial strategies and societal recognition. Coalitions exercised variable entrepreneurial strategies to produce change in supporting their communities’ health care needs. This variability was largely reflective of differences in their level of development. However, despite these differences, a pervasive barrier across the coalitions was the perception of limited societal and institutional recognition. Coalitions felt this was a key and continuing barrier to their activities and the effects they could achieve. These two main themes are now discussed.

### Main Theme: Entrepreneurial actions and strategies to effect change

Coalitions engaged tirelessly in entrepreneurial strategies to address the inequitable services they faced in their rural communities. Their diverse strategies, intended to produce short-term gains included: securing quick wins, leveraging existing resources, and joining forces. The entrepreneurial strategies of coalitions with longer histories and experience, a governance structure, and a diversity of talent appeared to achieve greater gains than those of coalitions that were struggling to form and produce effects.

#### Securing quick wins

Securing quick wins was an entrepreneurial activity coalitions used to take advantage of opportunities presenting at just the right time that moved towards their broader goals in supporting their citizens’ health care needs. For example, one community had no physician. A neighbouring community’s physician alerted the coalition to a funding opportunity for a nurse-practitioner (NP). The coalition immediately submitted a proposal and were successful in obtaining a nurse-practitioner for their community.

It was a very opportunistic time, right … because it was when they were rolling off the NP for B.C. funding. So it had to be that time to … kind of make that work. But it definitely came from a need of a lack of services in the community, right?

Often, these quick wins drew upon existing resources and capabilities that were different from the initial goals of the coalition, but nonetheless produced desired effects. For example, one community was given a one-week locum physician who also was establishing a virtual medical practice at that time. The coalition immediately identified a quick win -- bringing this virtually delivered care to the community as a way of meeting their interim needs for primary care.

So we set up a mobile … a station, a virtual connection station … in our clinic every Friday morning to do virtual connection with our community. We did everything we could to make sure there was a doctor there at some time … all that … you know, the best we could do.

Fundraising and the use of media were other entrepreneurial strategies for several coalitions to support quick, immediate health care needs. For example, several coalitions took advantage of all possible opportunities at local venues, such as Farmer’s Market, to raise funds with considerable success. One coalition that was fundraising to purchase a health clinic commented, “And then, after the first day of the farmers’ market she [one of the board members) sends us an e-mail. She says, “Ya, the farmers market was pretty good today. I raised $5,575!” Media was also used to produce quick wins particularly in crisis situations that threatened existing health services. For example, a coalition used the media to rally support for a local demonstration to successfully avert the threat of closure of their local emergency department due to physician shortages.

#### Leveraging existing resources

Beyond these quick wins, many coalitions were adept at leveraging local resources and individual relationships to create or adapt a range of services for older adults in their communities. Physical and human resources were used to great advantage in ensuring and supporting health care services. Coalitions used physical resources to meet local health care needs. For example one coalition used existing health clinic space to expand services, such as foot care and respiratory therapy. Leveraging their space was seen as an opportunity to enhance awareness of their community clinic to ultimately effect more comprehensive coverage of their primary health care needs. As one member articulated, “And every time we bring in another activity, somebody else is made aware of the fact that this place exists.” It was common for coalitions to draw on human resources, such as their memberships' local talent and experience in areas such as community development, website development, or lab technology. For example, to pay for sustaining their health clinic, one coalition used the expertise of one of their members to contract and bring private lab services to rent space in the clinic. Although not entirely the purview of coalition members, another coalition spoke of neighbours helping neighbours to access health care, “We see our neighbours driving out and other neighbours driving the neighbours to go to health centres. We get phone calls. You know, we all pitch in and help with our neighbours.”

Knowledge and use of existing services and resources gave coalitions the ability to meet their community’s specific needs such as transportation. For example, one community tagged onto an existing transportation service to create a branch of the service that targeted seniors:

So, what we’ve really been successful at, I think, is sort of working with what already exists and adding a senior’s component … Versus creating a whole other situation. Like, one of the initiatives I was thinking of was with … to do with transportation and we have the Rideshare program that was pretty well-established, so instead of trying to create something else … We just created a page on rideshare for seniors … With a very small grant. It was $500 total … That we provided to them and they created a video and a button and a senior’s … Information page to encourage that. And that’s … that’s endured as well. We’ve stayed in contact with them.

#### Joining forces

Joining forces was another entrepreneurial activity that successfully overcame divisiveness, fragmentation, and competition and facilitated “a collective voice for seniors’ concerns and services.” Joining forces involved coalitions liaising with local organizations and key stakeholders (e.g., physicians, politicians) and coalitions in other communities. As one coalition noted, “We recognized, as this key group [coalition], that we needed to have a better link and communication with our community.” Joining forces often occurred organically to support gaps in health care needs. For example, one community group described active groups in their community working on many initiatives, such as dementia friendly neighborhoods and home care, that allowed for this natural support to occur, as one coalition participant acknowledged, “Because I think anything that we need … that we’re shy of, there’s a group in town that’s working on it” and “we can ask each other for support.” In particular, long-running and well-established coalitions joined forces once they discovered their complementary strengths to mutually advance each other’s health service needs.

In doing so, more established coalitions were entrepreneurial in building partnerships with key stakeholders that strengthened their efforts and produced effects (e.g. a physician). Joining forces capitalized on synergies that built capacity and allowed coalitions to accomplish more together than apart. For example, more established coalitions could support less developed coalitions in advancing initiatives, producing effects that had mutual benefit, as one coalition described,

There’s clout. There’s a common voice of senior services at this table, so if we recommend a project, that carries some weight. And also, even for a group that say doesn’t have great capacity … For it to partner with us. So, that our capacity can help make their funding proposal stronger and then we work together on the project. They take the lead but we continue to support it.

Joining forces also facilitated information sharing/exchange, stronger funding proposals, growing talent pools (e.g., web developers, project managers, etc), and support for strategies such as community consultations. One coalition lead elaborated the value of joining forces with a neighboring coalition,

We felt from the beginning that this is not only a [single community] issue … it’s a small rural community issue and we need to work together with the other communities and share information that we have and things that worked here that are shared at. So we do have a good relationship with an organization in [community name] and their mayor and council. We have regular contact with them too.

### Main Theme: Lack of recognition

Despite their persistent and sometimes successful efforts to address the inequity of health service access in their communities, pervasive across all the coalitions at various points in time was limited societal and institutional recognition. A key barrier to their strategies and the effects of those strategies, lack of recognition was expressed as not being heard and not being seen. Governance and accountability issues created by their respective municipal governments and the regional health authority exacerbated the coalitions’ lack of recognition.

#### Not being heard

Coalitions were viewed as providing a consistent voice for their groups and communities. Often the impetus for forming as coalitions was to gain voice and be heard. As one member expressed, “Gradually this is how [the coalition] got to be formed, because people here knew that we all needed to have a say.” Coalitions wanted to be heard on such matters as meeting the needs of their older demographic, navigating the health authority/organization, securing space/resources for services (e.g., additional examination room in a local clinic), programming, funding, and implementing health care model alternatives. Despite the need and desire to be heard, “not being heard” was common across coalitions, especially in their dealings with the health authority. Coalitions experienced not being heard when the health authority lacked a timely response to their expressed needs, often creating barriers to accessing decision-makers, or failing to consult with coalitions on important decisions.

Coalitions repeatedly shared the frustration of long delays in response to service needs which in many cases were near a crisis point, and in other cases where the creation and mobilization of alternative forms of service delivery were needing timely health authority support. In these and other cases, coalitions expressed concerns about the seeming lack of reaction from the health authority to the growing “gray wave” to be served by a delivery model that “is less and less and less effective.” There was a general consensus among coalitions of feeling dismissed by the health authority, as one participant elaborated, “Well, just not responding, putting it off, referring to somebody else, not helping us to determine who we should be talking to … Moving positions … moving positions and then we’re talking to someone else.” The effect of not being heard by the health authority and their lack of involvement in matters that affected health care service delivery in their community made them feel small, insignificant, and powerless, like “fish in the ocean” or like “David and Goliath.” A coalition lead described, “we’re small fish in the ocean of health and bureaucracy, nobody asks our opinion about relevant local issues, decisions are made outside our involvement.”

Difficulties accessing health authority managers and decision-makers compounded the coalitions’ experiences of not being heard. Coalitions did “not know who to go to in the system” or “who we should be talking to” when they needed support and when inside information was required to advance their health care priorities and goals (primary health care, physician recruitment, and transportation). As an outsider, one coalition lead commented that “the biggest frustration we have is the fact that we are never allowed to talk to anybody who could actually make a decision.” One coalition even described strong opposition by a former health authority executive who reportedly said, “over my dead body” to coalition requests for primary team-based care.

#### Not being seen

Coalitions had a litany of successes such as delivering a unique healthcare model (e.g., a solo NP practice in a community that had struggled to recruit a primary care physician), or owning and operating a health clinic. Despite this, coalitions described a lack of recognition when they were “not being seen.” Coalitions described not being seen when their successes in bringing services to their communities went unnoticed, and when their unique rural health and service needs were not acknowledged. This lack of recognition was a highly emotionally charged area for coalitions since they believed that their efforts and successes made it possible for their local health care needs to be met. Coalitions believed their innovative models of primary health care warranted local and provincial recognition; locally to appreciate members for “everything that they’re doing”, and at the Ministry level as exemplars for how primary care could be delivered in rural areas.

Coalitions also experienced a lack of recognition for the unique nature, challenges and complexity of their efforts in promoting rural health care needs and services outside the relative understanding and ease of urban settings. These urban assumptions failed to account for rurality, and a system which simply considered “rural as mini-urban.” Although there was talk from the health authority about the special nature of rural health care, coalitions described a lack of care and follow-through from the “system”, as one coalition member captured,

“We were formed with the idea that we were going to come up with a model for rural health care because it’s just simply not the same, and although we get a whole lot of lip service from the system and we’re going to do this and we’re going to do that but nothing is happening. Nothing is happening.”

Coalitions identified the need and challenges of fitting an urban model into a rural context in terms of undermining their health care agendas, as one member so aptly described, “Our structures, which we take for granted, can defeat us in achieving these agendas. And basically reusing urban structures to kind of deal with rural situations.” Coalitions further experienced this lack of recognition when the system failed to “understand the value and expertise in rural communities”.

### Barriers and facilitators to recognition

Local/municipal government and health authority governance and accountability were influences on coalition recognition. Governance and accountability either created barriers or facilitated coalitions efforts in establishing themselves as recognized and legitimate participants within the healthcare system.

#### Governance

Local government structures created or eliminated certain barriers to action by community coalitions. For example, municipal government structures included incorporated, unincorporated and part of an electoral district, or other structures, such as an improvement district. Incorporated communities had greater formal local governing structures in place than unincorporated communities that facilitated a collective voice in advocating for health care services. Where these formal structures were absent, numerous divisive voices appeared and remained because of a lack of formal systems for strategizing and planning. A participant from one of the least developed coalitions described the governance situation in their community:

It is an improvement district with very limited capacity to govern anything. And I really see … you know, the gap that occurs in a place like this. There are some amazing things that occur because it is not a municipality … But there is absolutely no capacity to make plans … Community comes together in a crisis but can’t seem to come together in a structure, planned way to carry things out in a systematic manner, the divisions come into play again …

All coalitions experienced health authority bureaucratic structures, often characterized by opaque branches and silos, and complexity and variations in protocols, which constrained their ability to make their agendas and needs known. For example, the constant and frustrating changes and turnovers in health authority leadership and management stifled coalition voice and communication of their agenda, as one participant passionately conveyed:

… to define … who is the boss? You know, you just can’t figure it out. And then, there’s the manager of the manager of the manager and then the manager changes and they go … we’ll get back to you once we get a new manager and then that manager is doing two jobs. And so, okay … well, we’ll wait until you get it sorted out. In the meantime, we’re going to just go ahead with what we’re doing here.

Restructuring in the health authority left communities uncertain and silenced about what services they would have, and often created tensions between communities. One coalition participant described the uncertainty precipitated by restructuring:

When there’s this big changeover, the communities are kind of left hanging and we may not get those services back, literally. We may have them for several years and there’s a big rearrangement of everything and we may not get those services back or they may be reduced or … you know.

Coalitions described a history of restructuring and shifts in health care services that had served to suppress community voice. For example shifts from decentralized, local control to centralized control had long lasting effects on rural communities, as one coalition lead so poignantly captured:

With the closure of the Community Health Boards, it took the community voice totally out of health care and it has been out of it since then. And we are suffering the results of it and I believe that it’s fairly safe to say that even the people at the top know it isn’t working.

Restructuring often involved some communities gaining services and some losing them, with coalition members describing Health Authority use of divide and conquer approaches, pitting community against community which made developing a broader rural coalition agenda difficult.

#### Accountability

Coalitions routinely experienced a lack of organizational accountability from the health authority and local governments that precluded recognition. Coalitions described a lack of accountability with respect to transparency and participatory decision-making. The health authorities failure to provide accessible, timely information the coalitions required was a common occurrence. Simple requests for information to help them find people in decision making positions, through requests for such things as an organizational chart of the health authority, were never provided. As one coalition member lamented, “Our first request of [Health Authority] after we became a group was for an organizational chart. Okay. We still do not have one, “or “we asked for org chart and they all laughed. They said, “Try in a month or so.”

Coalitions often felt left out of decisions that directly affected them. Health authority decision-making that had a direct impact on their communities but did not take them into account, and failed to reflect their needs, wishes, or context, hindered coalition recognition. It was common for coalitions to describe changes to health services, such as emergency department closures or slowdowns or downgrading a community hospital to an ambulatory long-term care centre. Coalitions experienced top down decision-making without consultation as disrespectful, since they had knowledge of the local needs. As a member of a well-established coalition identified,

I think a lot of the issues are just resources and also maybe larger organizations and government and whoever makes decisions from the top down instead of actually consulting with the communities. There are people working in the communities that know what’s going on. They know what the needs are.

Political players at any given time influenced coalition recognition. Some municipal leaders were highly supportive and visionary, initiating action to advance services and to make community needs known, while others were slower to respond and required more prodding; “There is support from our present council when it’s pointed out to them that we need it. But the initiative is not there, and often there is a long struggle to get them onboard and convincing them.”

Provincial government mandates had left coalitions with assuming considerable responsibility for ensuring health services in their communities but without the concomitant authority and resources,. One coalition member lamented this constraint:

The Premier’s “Taxpayer Accountability” mandate has had the effect of downloading responsibility without either authority or resources. We believe we’ve gone as far as we can go without the involvement of paid professionals who can access “inside information” to ensure that the planning and development of our multi-community primary care network complies with both Provincial and Federal Government thinking.

Despite the barriers coalitions faced in being recognized for their entrepreneurial efforts, nevertheless in some cases there were facilitators to their recognition. For example, one coalition’s long history and efforts in engaging the health authority appeared to have produced longer-term recognition which had helped them make progress on a number of their health care service challenges. Numerous profitable encounters with the health authority had moved relationships along so that now they [health authority] were aware of the people and the roles. For example, working collectively as a group/coalition had produced success with referrals, and coalitions admitted feeling recognized and legitimate; “They’re aware of us now” and “a much better working relationship than we used to have.”

## Discussion

Coalitions’ required use of a number of entrepreneurial strategies led to modest health service delivery successes but it failed to address the broader recognition problem. This lack of recognition was also seen as a central barrier in coalitions’ consistent production of effects, and contributed to continuing inequities in health and health services faced by rural communities. These continuing inequities, often the result of organizational restructuring, commonplace across the Canadian health care system, intensified the work of the coalitions. The coalitions reported many thwarted attempts to make community needs known. They attributed the constraints they experienced in producing effects to the lack of recognition. This lack of recognition was experienced both within the community (e.g., municipal government) and even more so within the health authority.

This is one of the first studies to draw attention to recognition as a key barrier to coalition success even though recognition from other stakeholders has been identified as necessary for organizational success [23]. Lack of coalition recognition was similarly found in a recent study of 17 non-statutory organizations in the United Kingdom [24], in which organizations reported challenges in building and retaining partnerships with statutory organizations. They perceived that statutory organizations lacked awareness of their existence and understanding of their expertise, didn’t engage in two-way attempts to improve the situation, and supported effective information exchange to formal mechanisms only. In the current study, coalitions found it difficult to be treated as invisible when their efforts were so vital to meeting their community’s health care needs.

Recognition is a concept that identifies the ways in which people are dialogically constituted, and their identities shaped by the reflections of themselves received from others [25]. Recognition can be understood as the acknowledgment of a group or individual on their own terms, free from assumptions or false images projected onto them by others. Charles Taylor [25] describes recognition as “a vital human need, as well as something that is necessary for a flourishing democratic society” (pg. 47). Withholding recognition from an individual or a group is itself a form of oppression; non-recognition occurs when individuals or groups are “rendered invisible by authoritative institutions” [26]. In the context of current findings, recognition underpinned the struggle for coalitions to be acknowledged as having legitimate knowledge about their own circumstances, as well as the creativity and competence to cooperate as equal partners in addressing the health service inequities they faced.

Recognition within Nancy Fraser’s [27] conceptualization of social justice, has to do with determining political obligations, or who is to be formally acknowledged as a political subject, citizen, equal, and what form that recognition takes within our society. It is balanced with justice as *redistribution* or determining which goods ought to be distributed. Access to health care is about both distribution and recognition, however recognition is frequently overlooked in discussions of health equity or social justice. While a 2012 comparative analysis of public health policies in British Columbia and Ontario emphasized that reducing health inequities was a priority for British Columbia, the social justice issues that were identified were all quantifiable resource-based issues such as poverty and income inequality [28]. Rural communities face challenges of access in ways that create injustices with regard to both distribution and recognition [26]. Furthermore, it is not immediately evident from our findings that one has a causal effect on the other. Our findings demonstrate that coalition members directly identified both lack of resources and recognition; coalitions frequently voiced frustration not only about having less resources than they needed, but also not being heard or seen as a group trying to legitimately affect change.

Findings uncovered coalition members’ high level of understanding of the complexity of the health challenges that face rural communities. Coalitions’ explicit frustrations about not being heard or seen reflected their understanding that the health service inequities faced by their communities were not only from having less services, but also from a *felt* lack of recognition by those responsible for delivering services and allocating resources. In many cases, the lack of recognition added to existing access challenges by making it difficult for government and the health authority to understand the issues of inequitable distribution. Despite communicating their needs and developing enterprising solutions within their communities, many coalition members noted relevant institutions as unable/unwilling to recognize and hear the actual needs of their communities. Furthermore, coalitions described ways in which they were subject to a kind of exclusion, whereby the changes that they did effect were disregarded as legitimate solutions within the healthcare system. Though there were noted variations across the coalitions, many coalitions identified ways in which they felt they were unable to operate as a partner within the healthcare system.

The inability to achieve recognition from local or provincial government or from the health authority is not only a barrier to achieving health equity within rural communities but may also be a barrier to coalition entrepreneurship strategies. Rural health inequities might push communities towards these entrepreneurial strategies, but without recognition, these strategies may have difficulty taking hold, and may undermine a community or coalition’s ability to be entrepreneurial at all. The fatigue and frustration expressed by coalitions, able to make progress within their communities but not have these successes acknowledged or taken up by the health authority, was wearing on them as they pursued their goals. Finally, in cases where coalitions were able to achieve some results through entrepreneurial actions, however, it may not by itself necessarily produce a solution to rural health inequities. To argue more critically, the burden on communities to be entrepreneurs in order to achieve a similar access to health care as their urban counterparts may itself be seen as inequitable. Difficult access to the scarce resources required to innovate in rural settings, to eventually improve their health care situation, is an inequity compared to the resources others may have within reach.

Beyond equity to services or resources, or perhaps a cause of them, there is also inequity in the absence of institutional structures in rural settings, necessary for resource allocation for both health care services and entrepreneurial strategies. Young [29] defines justice as a concept that applies to *institutional conditions*, not only to the distribution of resources which may be a symptom of these conditions. When the focus of the coalition is on its effects on resource allocation, it misses attending to the conditions that determine and entrench inequitable or unjust resource allocations in the first place. Current findings demonstrate that in the perception of the coalitions there is a substantive difference between the ability to achieve effects and the ability to achieve recognition. Findings from this study suggest that even in instances where coalitions had achieved notable successes by way of increasing health services in their communities, those successes often went unnoticed or were not valued. What needs to be equitable, in our view, is not just the resources that rural communities have to address their health care needs, but the structural and institutional conditions under which those resources are distributed. Coalitions may have a role to play in drawing attention to the structural inequities, but it is a tall order to do so when they are caught up in constantly reacting to new crises to health service and resource inequities, and while being unable to speak to institutional conditions which may be beyond their time and capability for comment.

## Conclusion

Much of the literature on rural healthccare focuses on the increasingly urgent crises facing rural communities [30], whereas a majority of the literature on health coalitions addresses strategies outside of rural areas [20]. In examining rural coalitions’ challenges and successes, we uncovered the entrepreneurial strategies that coalitions and rural communities undertook to innovate and adapt with limited resources to improve material conditions and increase access to health services. However, we found that even if coalitions are able to effectively access health services through entrepreneurial strategies, it does not alleviate resource inequities, nor the institutional conditions that surround both health service and resource inequities. Research can continue to advance recognition as important to the entrepreneurial strategies of coalitions and their legitimacy. At the same time equal attention must be given to the institutional conditions that reinforce the inequities, as sole focus on coalition entrepreneurial strategies keeps the responsibility for accessing health care on the shoulders of rural citizens.

## Data Availability

The datasets generated and/or analysed during the current study are not publicly available because consent was not obtained from participants for this purpose.

## Abbreviation

CLC: Citizen-led coalitions
